# The Origin, Dynamic Morphology, and PI4P-Independent Formation of Encephalomyocarditis Virus Replication Organelles

**DOI:** 10.1128/mBio.00420-18

**Published:** 2018-04-17

**Authors:** C. E. Melia, H. M. van der Schaar, A. W. M. de Jong, H. R. Lyoo, E. J. Snijder, A. J. Koster, F. J. M. van Kuppeveld, M. Bárcena

**Affiliations:** a Department of Cell and Chemical Biology, Section Electron Microscopy, Leiden University Medical Center, Leiden, The Netherlands; b Department of Infectious Diseases and Immunology, Virology Division, Faculty of Veterinary Medicine, Utrecht University, Utrecht, The Netherlands; c Department of Medical Microbiology, Molecular Virology Laboratory, Leiden University Medical Center, Leiden, The Netherlands; Vanderbilt University Medical Center

**Keywords:** cardiovirus, picornavirus, replication organelles, electron tomography, membrane structure, PI4P, phosphatidylinositol 4-kinase type III alpha, electron microscopy, phospholipids

## Abstract

Picornaviruses induce dramatic rearrangements of endomembranes in the cells that they infect to produce dedicated platforms for viral replication. These structures, termed replication organelles (ROs), have been well characterized for the *Enterovirus* genus of the *Picornaviridae*. However, it is unknown whether the diverse RO morphologies associated with enterovirus infection are conserved among other picornaviruses. Here, we use serial electron tomography at different stages of infection to assess the three-dimensional architecture of ROs induced by encephalomyocarditis virus (EMCV), a member of the *Cardiovirus* genus of the family of picornaviruses that is distantly related. Ultrastructural analyses revealed connections between early single-membrane EMCV ROs and the endoplasmic reticulum (ER), establishing the ER as a likely donor organelle for their formation. These early single-membrane ROs appear to transform into double-membrane vesicles (DMVs) as infection progresses. Both single- and double-membrane structures were found to support viral RNA synthesis, and progeny viruses accumulated in close proximity, suggesting a spatial association between RNA synthesis and virus assembly. Further, we explored the role of phosphatidylinositol 4-phosphate (PI4P), a critical host factor for both enterovirus and cardiovirus replication that has been recently found to expedite enterovirus RO formation rather than being strictly required. By exploiting an EMCV escape mutant, we found that low-PI4P conditions could also be overcome for the formation of cardiovirus ROs. Collectively, our data show that despite differences in the membrane source, there are striking similarities in the biogenesis, morphology, and transformation of cardiovirus and enterovirus ROs, which may well extend to other picornaviruses.

## IMPORTANCE

Like all positive-sense RNA viruses, picornaviruses induce the rearrangement of host cell membranes to form unique structures, or replication organelles (ROs), that support viral RNA synthesis. Here, we investigate the architecture and biogenesis of cardiovirus ROs and compare them with those induced by enteroviruses, members of the well-characterized picornavirus genus *Enterovirus*. The origins and dynamic morphologies of cardiovirus ROs are revealed using electron tomography, which points to the endoplasmic reticulum as the donor organelle usurped to produce single-membrane tubules and vesicles that transform into double-membrane vesicles. We show that PI4P, a critical lipid for cardiovirus and enterovirus replication, is not strictly required for the formation of cardiovirus ROs, as functional ROs with typical morphologies are formed under phosphatidylinositol 4-kinase type III alpha (PI4KA) inhibition in cells infected with an escape mutant. Our data show that the transformation from single-membrane structures to double-membrane vesicles is a conserved feature of cardiovirus and enterovirus infections that likely extends to other picornavirus genera.

## INTRODUCTION

The cardioviruses comprise an important genus of the *Picornaviridae* family of positive-sense RNA (+RNA) viruses. The type species *Cardiovirus A*, or encephalomyocarditis virus (EMCV), ostensibly infects mice but is capable of infecting a range of mammals, including humans. Severe disease in wild or farm animals occurs primarily in swine (1, 2), but concerns for human safety have followed from studies demonstrating the potential for pig-to-human EMCV transmission during organ xenotransplantation (3, 4), which will become more imminent as the technique matures. The *Cardiovirus B* species is represented by Theiler’s murine encephalomyocarditis virus (TMEV), the study of which has led to critical contributions to our understanding of demyelinating diseases (reviewed in reference 5). Unlike TMEV, the closely related Saffold virus is capable of infecting humans. Saffold virus was discovered only recently in a patient with a fever of unknown origin (6) but appears to be widespread and commonly found in coinfections with other viruses. Among these coinfecting viruses are the enteroviruses (7, 8), another genus of picornaviruses that includes poliovirus, the coxsackieviruses, rhinoviruses, and enteroviruses A71 and D68. Although the consequences of enterovirus and Saffold virus infections in coinfected patients are entwined, there are suggestions that Saffold virus could cause or contribute to diseases as diverse as gastroenteritis, encephalitis, myocarditis, and nonpolio acute flaccid paralysis (reviewed in reference 9), which are typically associated with enterovirus infections.

Understanding the conserved requirements for picornavirus replication is an important strategy for developing broadly acting antiviral therapies. Currently, vaccines are available to prevent infections with hepatitis A virus, poliovirus, and enterovirus 71 (EV71) (available only in China), but there are no approved antiviral therapies to treat picornavirus infections. Substantial efforts have been invested in developing directly acting antivirals that target viral proteins, but the spectrum of antiviral activity of these agents is limited (10–12). Another strategy, for which interest has been growing in recent years, is the development of inhibitors targeting host factors that are conserved among different genera (13). One host factor required for the replication of both enteroviruses and cardioviruses is the lipid phosphatidylinositol 4-phosphate (PI4P). Cellular production of PI4P is orchestrated by phosphatidylinositol 4-kinases (PI4Ks), which reside in various subcellular compartments and generate local pools of PI4P (reviewed in references 14 and 15). Enteroviruses rely upon the III beta class of PI4K (PI4KB) (16, 17), which predominantly localizes to the Golgi apparatus, while cardioviruses require the largely endoplasmic reticulum (ER)-based III alpha class of PI4K (PI4KA) (18). Interestingly, as with cardioviruses and enteroviruses, PI4P is also required by the more distantly related hepatitis C virus (HCV), which depends upon PI4KA for its replication (19).

A role for PI4P in expediting the formation of enterovirus replication organelles (ROs) has recently been described (20), which could in part explain its importance during viral replication. ROs are virus-induced membrane rearrangements formed during infections of all eukaryote-infecting +RNA viruses. These structures serve as a hub for viral replication in the cell, and their unique properties are believed to confer intrinsic benefits on the viruses that generate them. The expansion of membranes that support the viral RNA synthesis machinery may increase replication efficiency, while the unique morphology of some ROs could serve to compartmentalize this process or its products and limit host innate immune sensing. The three-dimensional (3D) morphology of enterovirus ROs is well characterized. Early in infection, enteroviruses induce the formation of single-membrane tubular ROs that gradually transform into double-membrane vesicles (DMVs) and multilamellar vesicles over the course of infection (21, 22). Our understanding of cardiovirus ROs, however, is more limited. Different studies have identified single- or double-membrane structures using two-dimensional (2D) electron microscopy (EM) of chemically fixed samples (23–25), and it is unclear whether these disparate observations reflect differences in sample preparation or truly distinct structures, perhaps found at different stages of infection. It remains to be established how membrane modifications arise and develop across cardiovirus infection, what their 3D morphology is, which structures are capable of supporting RNA synthesis, and, furthermore, whether PI4KA plays a role in cardiovirus RO formation.

Here, we use serial electron tomography (ET) to unravel the 3D morphology of EMCV ROs across an extensive time course that encompasses their formation and development. Samples were prepared using high-pressure freezing and freeze substitution (HPF-FS) as an alternative to chemical fixation, which better preserves structures sensitive to artifacts. We provide evidence that early EMCV ROs consist of single-membrane tubules (SMTs) and vesicles (SMVs) that emerge from the ER. These single-membrane structures, which arise during the exponential phase of viral RNA synthesis, appear to serve as the primary platform for genome replication. However, double-membrane vesicles (DMVs), which largely emerge later in infection, were also capable of supporting RNA synthesis. Remarkably, putative transition states between single-membrane tubules and DMVs were found that mirror the transformation intermediates observed for enterovirus ROs (21). This chronological progression of RO morphology contextualizes the previous observations of single- or double-membrane EMCV ROs and represents a striking parallel between cardiovirus and enterovirus DMV formation that may represent a conserved mechanism among picornaviruses. Given this cohesion in RO morphology between enteroviruses and EMCV, we then investigated whether PI4KA plays a role in the formation of EMCV ROs, as PI4KB does for enterovirus ROs (20). To this end, we studied the effects of PI4KA inhibition on an EMCV mutant that harbors a single substitution in its 3A protein (3A-A32V), which confers resistance to PI4KA inhibitors (26). Interestingly, PI4KA inhibition neither affected RO morphology nor significantly delayed RO formation. This indicates that, similarly to enteroviruses, a lack of high PI4P levels does not pose an unsurmountable barrier for cardiovirus RO formation.

## RESULTS

### EMCV-induced membrane rearrangements with different morphologies arise over the course of infection.

**FIG 1:**
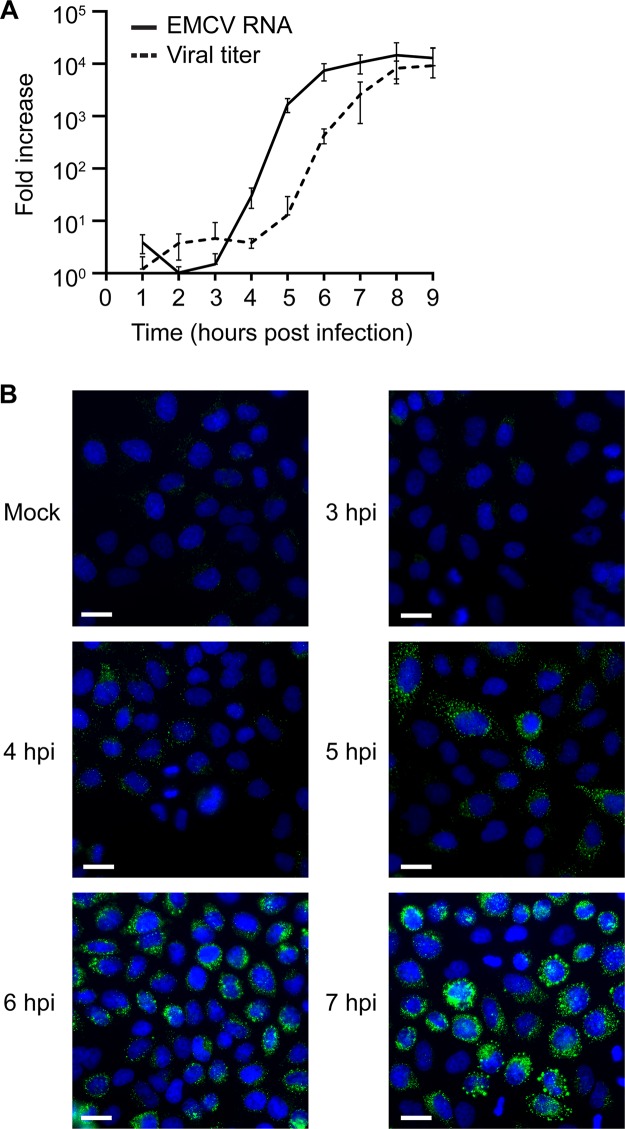
The kinetics of EMCV replication and progeny virus production. HeLa cells were infected or mock infected with EMCV. (A) Cells were lysed every hour between 1 and 9 hpi to determine viral RNA levels by qPCR or to determine viral titer by titration. Values, converted to fold increase, represent the mean from triplicates (± standard error). (B) The subcellular distribution of dsRNA (green) relative to the cell nucleus (blue) was assessed by immunofluorescence analysis every hour between 3 and 9 hpi (3 to 7 hpi shown). Bars, 20 µm.

We first set out to determine the relationship between EMCV replication and the emergence of ROs. Samples for quantitative PCR (qPCR), viral titer determination, immunofluorescence analysis, and electron microscopy (EM) (chemical fixation) were generated within a single experiment. HeLa cells infected with EMCV (strain Mengovirus) at a multiplicity of infection (MOI) of 50 were processed every hour up to 9 h postinfection (hpi) for qPCR of viral RNA and for the determination of viral titer. Samples for immunofluorescence and electron microscopy (EM) analyses were fixed between 3 and 9 hpi. While data from these chemically fixed EM samples were assessed to quantify the emergence of ROs, representative images are presented from a subsequent experiment using an improved sample preparation method (high-pressure freezing and freeze substitution) that more faithfully preserves RO morphology. The exponential phase of RNA synthesis spanned 3 to 6 hpi, while the increase in viral titer lagged by ~2 h (Fig. 1A). These RNA replication dynamics aligned well with increasing double-stranded RNA (dsRNA) immunofluorescence signal, which provides a secondary indication of viral replication. The first fluorescent signal was detected at 4 hpi, and levels increased steadily each hour before reaching a plateau at 7 hpi. Between 4 and 5 hpi, dsRNA signal was found at small foci distributed throughout the cytoplasm, which became larger and more clustered as infection progressed (Fig. 1B).

**FIG 2:**
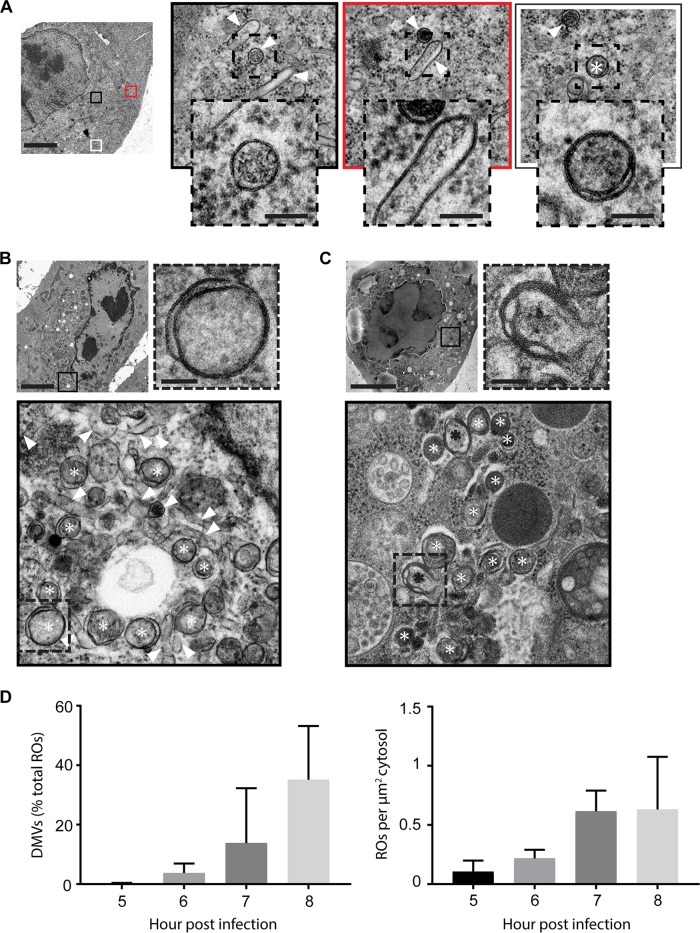
2D EM reveals single-membrane, double-membrane, and multilamellar structures. An initial 2D EM analysis to assess the emergence and development of EMCV ROs was performed using chemically fixed HeLa cells processed in parallel with samples described for Fig. 1 (D). Corresponding structures in high-pressure frozen and freeze-substituted samples are shown (A to C). (A) Membrane rearrangements found at early infection time points (~5 hpi) were primarily single-membrane structures (arrowheads), but some DMVs (white asterisks) were also observed. (B) Later in infection (~6 hpi), the membrane rearrangements became more clustered and the relative abundance of DMVs increased. (C) At late infection time points (~7 hpi), double-membrane structures were often predominant and multilamellar membrane structures were found (black asterisks). (D) Relative abundance of DMVs in EMCV-infected chemically fixed cells between 5 and 8 hpi (left). Increase in total ROs over this period is expressed as RO counts per µm^2^ of cytoplasm (right). Total RO count from 5 to 8 hpi = 4,365; 5 cell sections randomly selected and analyzed per time point. The bars represent standard deviations. (A to C) Bars, 5 µm (unboxed images) or 100 nm (dashed-line boxed images).

The first virus-induced membrane modifications were detected in EM cell sections of cells fixed at 5 hpi, revealing a complex but sparse assortment of virus-induced single-membrane structures (Fig. 2A, arrowheads) and, very occasionally, double-membrane structures (Fig. 2A, asterisk). From 6 hpi, membrane modifications became increasingly clustered into large regions that dominated the cytosol, and the abundance of double-membrane structures increased (Fig. 2B, asterisks), although single-membrane structures persisted (Fig. 2B, arrowheads). Late in infection, double-membrane vesicles became more dominant (Fig. 2C, white asterisks), and multilamellar structures were also found (Fig. 2C, black asterisks and inset). Between 5 and 8 hpi, the RO density in the cytoplasm greatly increased (from 0.11 to 0.63 ROs per µm^2^), and the proportion of DMVs (as a percentage of total ROs) grew from less than 1% to an average of 35%, although in some cells DMVs rather than single-membrane structures were the predominant RO morphology (Fig. 2D). A number of cells with evident signs of lysis (e.g., disrupted plasma membrane and extracted cytosolic content) could also be found later in infection, which may explain the lack of immunofluorescence labeling in some cells by 7 hpi.

### Electron tomography reveals the 3D architecture and transformation of ROs.

One drawback of 2D EM cell section analysis is the similarity in cross section between vesicles and tubules running perpendicular to the section plane. Additionally, features like membrane connections or small openings may be obscured by the superposition of structures in a 2D projection image. To more unambiguously characterize the morphology of EMCV ROs, cells at early and late stages of infection were prepared for electron tomography (ET). Tomograms from serial cell sections of the same region of interest were combined to form large 3D volumes.

**FIG 3:**
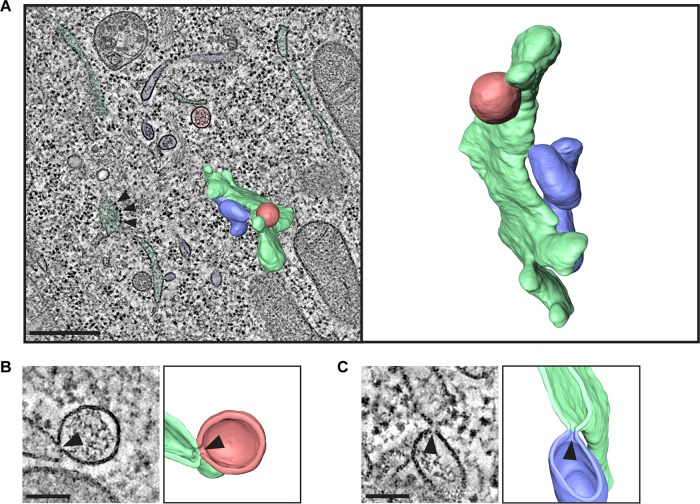
Tomography of EMCV-infected cells at 5 hpi reveals connections between single-membrane ROs and ER. HeLa cells were infected with EMCV and high pressure frozen at 5 hpi for EM processing and serial tomography. Shown are different features observed in a serial tomogram comprising five consecutive sections of 200-nm thickness from a representative cell. (A) Section through the tomogram volume showing SMVs in red, SMTs in blue, and ER in green, recognizable by the membrane-associated ribosomes (arrowheads), with a segmentation of a subregion of the volume superimposed (left) and in isolation (right). (B and C) Sections through the tomogram alongside their corresponding 3D model cutaways reveal connections between SMVs (red) and ER (green) (B) and between SMTs (blue) and ER (green) (C). The site of the connection is indicated with a black arrowhead. Bars, 500 nm (A) or 100 nm (B and C).

**FIG 4:**
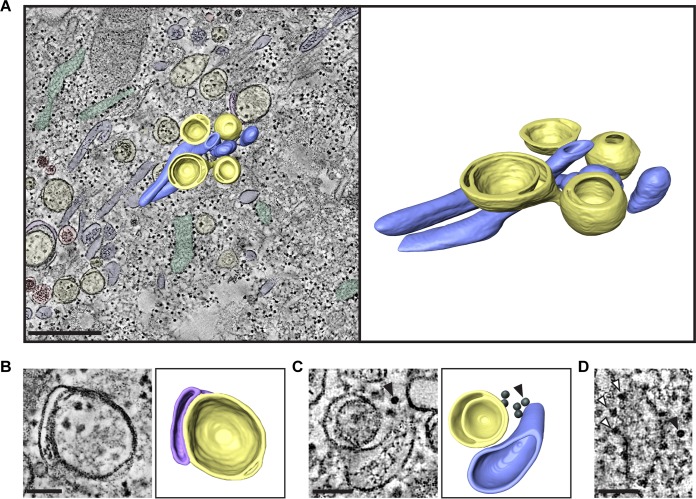
Tomography of EMCV-infected cells at 7 hpi highlights virions proximal to DMVs and multilamellar vesicles. HeLa cells were infected with EMCV and high pressure frozen at 7 hpi for EM processing and tomography. Shown are structures observed in a serial tomogram comprising two consecutive sections of 200-nm thickness from a representative cell at this time point. (A) Section through the tomogram and corresponding model showing SMTs in blue, SMVs in red, ER in green, DMVs in yellow, and cisternae enwrapping DMVs in purple, with a segmentation of a subregion of the volume superimposed (left) and in isolation (right) highlighting SMTs and DMVs. (B and C) Sections through tomograms and corresponding models showing a multilamellar vesicle, which consists of a closed DMV (yellow) enwrapped by a flattened cisterna (purple) (B), and highlighting the proximity of viral particles (black) in the cytosol to ROs (yellow and blue) (C). Virus particles are indicated with an arrowhead. (D) Comparison of ribosome (white arrowheads) and virus particle (black arrowhead) morphology in a single tomogram section. Bars, 500 nm (A) or 100 nm (B to D).

Single-membrane structures identified in the 2D analysis at early times postinfection were found to comprise a mixed population of single-membrane vesicles (SMVs) and single-membrane tubules (SMTs) (Fig. 3A, red and blue, respectively; see also Movie S1 in the supplemental material). Unlike the tubules formed during enterovirus infections, EMCV SMTs did not form tightly packed clusters. Both SMVs and SMTs were smooth-membrane structures and were frequently found connected to rough ER (green) by neck-like membrane connections (Fig. 3B and C, arrowheads), like the ER-derived ROs of nidoviruses (27–29) and HCV (30). In contrast, DMVs, which were predominantly found at late stages of infection, appeared in all instances as separate compartments, with no membrane connections to other structures (Fig. 4A, yellow; Movie S2). Some DMVs seemed to be partially enwrapped by additional cisternae late in infection (Fig. 4B, purple), to form multilamellar structures. Virus particles were also detectable in the EM data as dense hexagonal profiles of ~25 nm (Fig. 4C, arrowhead), which were frequently found in the cytosol within 30 nm of ROs (Fig. S1). These particles (Fig. 4D, black arrowhead) were distinguishable from ribosomes (Fig. 4D, white arrowheads) by their symmetry and well-defined edges. A closer spatial association was found between virus particles and RO membranes (SMVs, SMTs, or DMVs) than between randomly generated coordinates and RO membranes (Fig. S1, *P* < 0.001). This could reflect localized clustering of virions following their formation or a spatial connection between RNA synthesis and virion assembly.

10.1128/mBio.00420-18.1 FIG S1  Analysis of the distribution of virus particles shows an association between EMCV virions and ROs. Distribution of EMCV virions relative to nearest RO membrane in HeLa cells infected with EMCV at 7 hpi (pooled data from two tomograms from two different cells). The distances from the center of virions to the nearest SMV, SMT, or DMV RO membrane were measured (*n* = 183, represented in upper graph). To independently assess whether this measured distribution of particles relative to ROs differed from a random distribution, tomograms were populated with models of simulated particles by randomly generating coordinates within the cytosolic space of the tomogram volume. The distances between the centers of these randomly generated particles and the nearest RO membrane were then measured (*n* = 183, represented in lower graph). The cumulative frequency distributions of the observed and generated data were found to be significantly different (Kolmogorov-Smirnov, *P* ≤ 0.0001). Download FIG S1, TIF file, 0.3 MB. Copyright © 2018 Melia et al. 2018 Melia et al. This content is distributed under the terms of the Creative Commons Attribution 4.0 International license.

10.1128/mBio.00420-18.4 MOVIE S1  Reconstructed and joined serial tomograms and segmentation of ROs found at early stages of EMCV infection. The complete tomogram volume (1 µm by 2.4 µm by 2.4 µm) of the region featured in Fig. 3A is shown, which represents a typical region in an infected cell at 5 hpi. Segmentation of a subvolume within these data highlights connections between SMTs (blue) or SMVs (red) and the endoplasmic reticulum (green). Download MOVIE S1, AVI file, 18 MB. Copyright © 2018 Melia et al. 2018 Melia et al. This content is distributed under the terms of the Creative Commons Attribution 4.0 International license.

10.1128/mBio.00420-18.5 MOVIE S2  Reconstructed and joined serial tomograms and segmentation of ROs found at late stages of EMCV infection. The complete tomogram volume (0.4 µm by 2.3 µm by 2.3 µm) of the region featured in Fig. 4A is shown, which represents a typical region in an infected cell at 7 hpi. Segmentation of a subvolume within these data highlights clusters of DMVs (yellow) and SMTs (blue). Download MOVIE S2, AVI file, 19.1 MB. Copyright © 2018 Melia et al. 2018 Melia et al. This content is distributed under the terms of the Creative Commons Attribution 4.0 International license.

**FIG 5:**
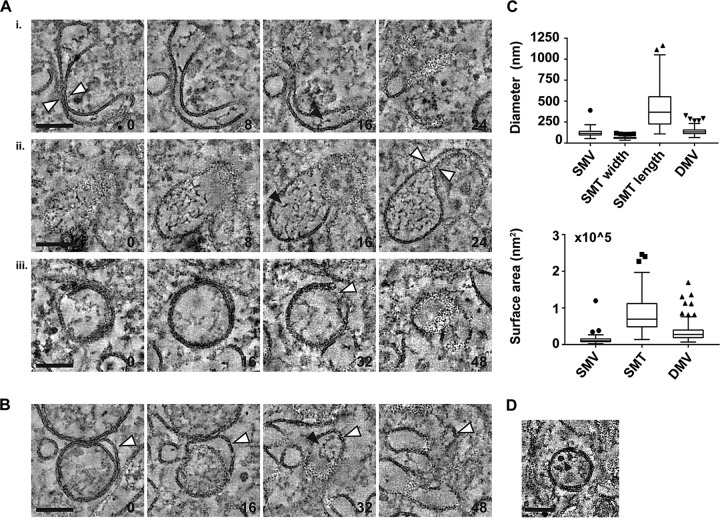
Intermediate structures suggest that single-membrane structures transform into DMVs. Intermediate structures found in EMCV-infected HeLa cells at 7 hpi. The slice thickness is 1.24 nm, and the slice spacing (A and B) is indicated in the lower right corner of consecutive images. Black arrowheads indicate electron-dense material present in the lumen of SMTs and between the inner and outer membranes of DMVs. (A) Membrane curving and pairing of single-membrane structures (white arrowheads, i and ii) and a DMV with an opening to the cytosol (white arrowhead, iii). (B) DMV with a tubular extension of its outer membrane (indicated by white arrowheads). (C) Tukey plots showing the distribution of diameters (top) and surface areas (bottom) of ROs. Median diameter values (nanometers) are 115 (SMV, *n* = 90), 62 (SMT width, *n* = 90), 368 (SMT length, *n* = 90), and 132 (DMV, *n* = 130). (D) Example of a virus particle (black arrowhead) enclosed within a DMV. Bars, 100 nm.

Ultrastructural investigations of enterovirus ROs have revealed putative transition structures that may represent the transformation of SMTs into DMVs. This process appears to involve the pairing of membrane tubules to form flattened cisternae, which curve to enwrap a small volume of cytosol, ultimately forming an open DMV in a vase-like configuration. The opening then seals to form a complete, closed DMV (21, 22). Similar structures were found in tomograms of EMCV-infected cells, including paired and highly curved tubules (Fig. 5A, i and ii, white arrowheads), which could represent an early stage of DMV formation from SMTs, and DMVs with small openings (Fig. 5A, iii, arrowhead). Tubular extensions of the outer membranes of DMVs were also found (Fig. 5B, indicated by white arrowheads in consecutive sections) that could represent the partial transformation of SMTs to DMVs. Supporting this idea, similar electron-dense material was observed both in the lumen of single-membrane structures (Fig. 5A, black arrowheads) and within the intermembrane space created by these tubular extensions (Fig. 5B, black arrowhead). To further examine the possibility of a single- to double-membrane structure transformation, the surface areas of SMVs, SMTs, and DMVs were estimated using the measured average sizes for each structure (Fig. 5C, top). These figures provide a guide as to whether SMTs or SMVs are likely candidate precursor structures for DMVs (as described in reference 21) as, unless other lipid sources contribute to DMV membranes, any precursor structure should have at least the same surface area as the DMV that forms from it. The estimated surface areas generated allow for a scenario where DMVs are formed by SMTs (Fig. 5C, bottom) and are compatible with the possibility that some (smaller) DMVs could also be derived from SMVs. In this scenario, the multilamellar vesicles seen at late time points may arise from SMTs that enwrap or partially enwrap existing DMVs, rather than enwrapping the cytosol to form a new DMV. DMVs were occasionally found to contain virions in samples analyzed at 7 hpi (pooled data from four tomograms; example shown in Fig. 5D) but at a low frequency (7% of 68 DMVs), and particles within DMVs represented a small fraction of total virus particles detected (2% of 339 particles). An enwrapping mechanism of SMT-to-DMV transformation could explain the presence of virus particles within a subset of DMVs, as virions may be enclosed by chance during the process.

### RNA synthesis occurs at virus-induced single- and double-membrane structures.

To investigate whether all or some of these virus-induced membrane modifications support RNA synthesis, metabolic labeling and EM autoradiography (31) were performed. Cells were infected with EMCV and fixed at 5 or 7 hpi. During the 2 h prior to fixation, cells were incubated with 10 µg/ml dactinomycin to limit cellular transcription. During the 45 min prior to fixation, cells were additionally treated with tritiated uridine to label newly synthesized RNA. After chemical fixation and preparation for EM, sections were processed for autoradiography.

**FIG 6:**
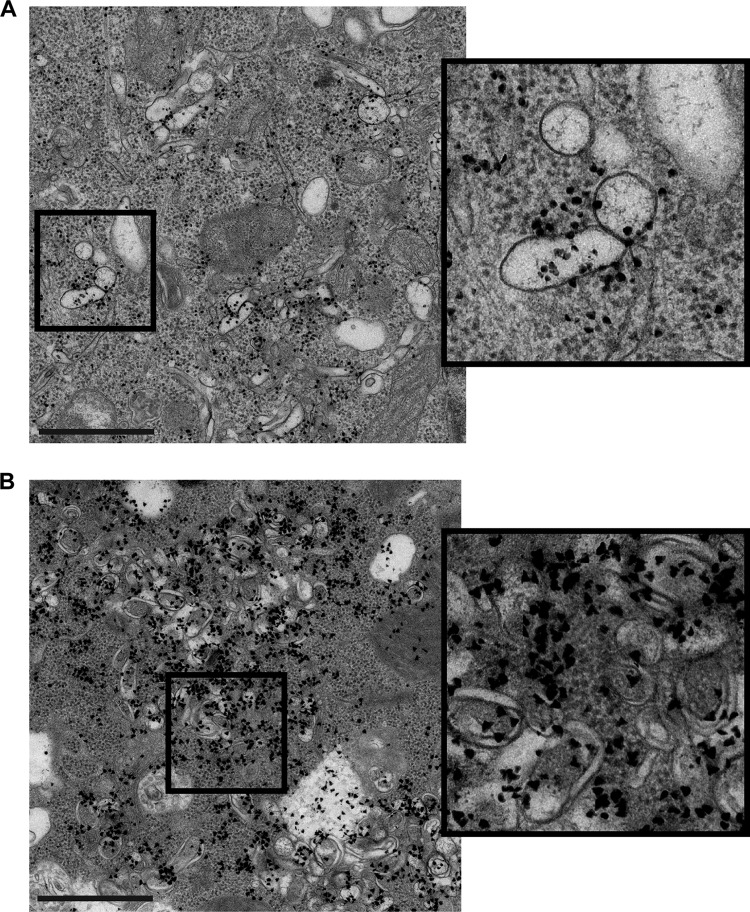
RNA synthesis occurs at single- and double-membrane structures. HeLa cells were infected with EMCV, metabolically labeled with tritiated uridine in the presence of dactinomycin, chemically fixed at 5 or 7 hpi, and processed for EM autoradiography to detect the subcellular location of newly synthesized viral RNA in 2D EM cell sections. (A) Cells at early stages of infection revealed autoradiography signal (electron-dense grains) clustered around areas with single-membrane structures. (B) In cells at late stages of infection, autoradiography signal was found at RO clusters primarily comprised of double-membrane structures. Bar, 2 µm.

While only larger clusters of electron-dense grains are good indicators of underlying viral RNA synthesis given the limited resolution of EM autoradiography (32), substantial autoradiography signal could be found clustered around areas containing exclusively single-membrane tubules and vesicles (Fig. 6A). This demonstrates their ability to support viral RNA synthesis. Newly synthesized RNA was also evident at RO foci comprised predominantly of DMVs at later stages of infection (Fig. 6B), indicating that the transformation from single-membrane structure to DMV does not impede continued genome replication.

### PI4KA inhibition of a PI4K-resistant EMCV mutant does not alter RO development or morphology.

While there may be divergence in the origins of enterovirus and cardiovirus ROs, the development of SMTs that transform into DMVs appears to be a common feature of both. We next investigated whether common requirements for RO biogenesis might also exist between enteroviruses and cardioviruses. Specific roles for the enterovirus host factor PI4KB, a PI4P-generating kinase, have recently been identified. In addition to facilitating efficient polyprotein processing (20, 33–35), PI4KB accelerates the formation of enterovirus ROs (20). To investigate possible roles during EMCV RO formation of PI4KA, an essential PI4P-generating kinase for cardiovirus replication, we utilized the 3A-A32V mutant (26). This mutant, generated through serial passaging of wild-type (wt) EMCV in cells with a stable knockdown of PI4KA, is capable of establishing replication even in the presence of the PI4KA inhibitor A1. At a concentration of 10 nM, this compound potently inhibits wt EMCV replication in HeLa cells, with no effect on cell viability (26).

**FIG 7:**
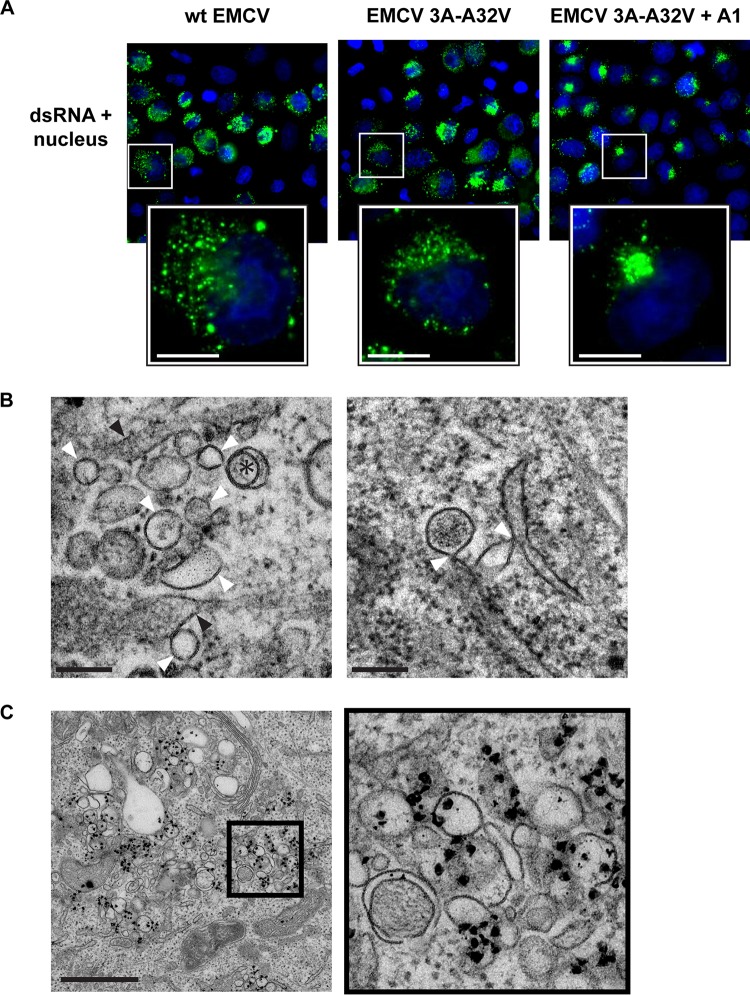
RO development during EMCV 3A-A32V infection under PI4KA inhibition. HeLa cells were infected with wt EMCV or EMCV 3A-A32V and fixed at 5 or 7 hpi. (A) Immunofluorescence labeling reveals a dense perinuclear distribution of dsRNA (green, nuclear staining in blue) in cells infected with EMCV 3A-A32V and treated with PI4KA inhibitor A1, compared to wt EMCV or EMCV 3A-A32V infections without inhibition (7 hpi). (B) Single-membrane structures (white arrowheads) and double-membrane structures (asterisk) could be found in infections with EMCV 3A-A32V under PI4KA inhibition that resembled those of wt EMCV (left, 7 hpi; black arrowheads, ER). Connections between single-membrane ROs and the ER (arrowheads) could also be found (right, 5 hpi). (C) Cells were infected with EMCV 3A-A32V, treated with PI4KA inhibitor, metabolically labeled, chemically fixed at 7 hpi, and processed for EM autoradiography to detect the subcellular location of newly synthesized viral RNA in 2D EM cell sections. Autoradiography signal (electron-dense grains) accumulated at RO clusters, similar to observations of wt EMCV infection. Bars, 10 µm (A), 200 nm (B), or 1 µm (C).

In the absence of A1 treatment, infections by the EMCV 3A-A32V mutant in HeLa cells produced ROs whose morphology was indistinguishable from those of wt EMCV (Fig. S2). SMTs and SMVs predominated at earlier time points, while DMVs were found only occasionally (Fig. S2A, asterisk). Later in infection, DMVs proliferated and ROs became more clustered (Fig. S2B, DMVs indicated by asterisks). Together, these results indicate that the 3A-A32V substitution does not affect RO development or the RO general architecture. PI4KA inhibition under A1 treatment led to a more clustered, perinuclear dsRNA signal compared to uninhibited infections with EMCV 3A-A32V or wt EMCV (Fig. 7A) (26). Despite this different staining pattern under PI4KA inhibition, the morphology of ROs and time postinfection at which ROs were first detected were not affected. EMCV 3A-A32V infection under PI4KA inhibition produced single- and double-membrane cardiovirus ROs (Fig. 7B, left, white arrowheads and asterisks, respectively), typically in proximity to the ER (black arrowheads). Importantly, while the clustered dsRNA signal found under inhibition partially localized in the perinuclear Golgi region (Fig. S3), connections between single-membrane ROs and the ER were still found (Fig. 7B, right, white arrowheads), and ROs produced under PI4KA inhibition also retained their ability to support RNA synthesis (Fig. 7C). Taken together, our data demonstrate that, while PI4KA/PI4P availability may govern the subcellular location of EMCV ROs, it does not significantly affect their emergence, morphology, or ability to serve as platforms for viral RNA synthesis.

10.1128/mBio.00420-18.2 FIG S2  The ROs formed by EMCV 3A-A32V are indistinguishable from those of wt EMCV. HeLa cells infected with EMCV 3A-A32V were frozen at 5 or 7 hpi for EM processing and 2D cell section analysis. The virus-induced single-membrane and double-membrane structures found at earlier (A) and later (B) stages of infection were morphologically identical to those formed during wt EMCV infection. Double-membrane structures are marked with a white asterisk. Bars, 5 µm (unboxed images) or 100 nm (dashed-line boxed images). Download FIG S2, TIF file, 2.5 MB. Copyright © 2018 Melia et al. 2018 Melia et al. This content is distributed under the terms of the Creative Commons Attribution 4.0 International license.

10.1128/mBio.00420-18.3 FIG S3  EMCV 3A-A32V dsRNA signal clusters in the perinuclear Golgi region under PI4KA inhibition. HeLa cells were infected with EMCV 3A-A32V at an MOI of 75 with or without 10 nM A1 treatment to inhibit PI4KA and fixed at 7 hpi. The *trans-*Golgi network marker TGN46 was labeled with a primary rabbit polyclonal antibody (Novus Biologicals) and secondary Alexa Fluor 594-conjugated goat anti-rabbit IgG for immunofluorescence analysis in a Leica SPII confocal microscope. PI4KA inhibition led to clustering of dsRNA signal (green) around the perinuclear region in foci that were often proximal to the TGN46 signal (red). Bar, 10 µm. Download FIG S3, TIF file, 1.3 MB. Copyright © 2018 Melia et al. 2018 Melia et al. This content is distributed under the terms of the Creative Commons Attribution 4.0 International license.

## DISCUSSION

Here, we reveal the 3D morphology of cardiovirus ROs and present a comprehensive overview of their formation and development throughout infection. At early stages of EMCV infection, virus-induced SMTs and SMVs were found, which appear to transform into DMVs over the course of infection. This progression bears a striking resemblance to that of enterovirus ROs, suggesting a universal mechanism for DMV formation in picornaviruses. Despite differences in the cellular origins of enterovirus and cardiovirus ROs, our data establish that escape mutants from both genera are able to bypass any role of PI4P in RO formation.

Single-membrane ROs which were capable of supporting viral RNA synthesis predominated at early stages of EMCV infection. Interestingly, while enteroviruses invariably produce SMTs early in infection (21, 22), also in HeLa cells (22), EMCV produced both SMTs and SMVs. These structures were of different sizes and did not form packed clusters like the SMTs of enteroviruses. Many of the SMTs and SMVs visualized at early stages of infection were also found connected to the ER, providing compelling evidence that cardiovirus ROs are ER derived. This finding is supported by the reported reliance of cardioviruses on ER-associated proteins like PI4KA and the observed colocalization of calreticulin and RO-associated viral proteins (18). Unlike cardioviruses, enteroviruses depend on Golgi apparatus-based proteins like PI4KB and GBF1 (16, 17, 36) and likely utilize Golgi membranes to generate their ROs. Interestingly, membrane connections between donor organelle and enterovirus ROs were not detected in the tomography studies (21, 22), suggesting that the process of initial enterovirus RO budding and release from the donor organelle is rapid compared to that of cardioviruses.

Despite these divergences in origin and morphology, our findings suggest that the transformation of single-membrane ROs into DMVs is conserved among enteroviruses and cardioviruses. For both genera, DMV formation appears to occur via membrane pairing of single-membrane ROs to form curved cisternae that ultimately enwrap small volumes of cytosol (21, 22). Given that transport from the DMV interior to the cell cytosol across the double membrane of a closed DMV is problematic, these structures may represent a means of sequestering viral products at late stages of infection. Interestingly, a small population of DMVs were also found with openings to the cytosol. While these open DMVs could reflect an intermediate step in their transformation from single-membrane structures to closed DMVs, it is also possible that a small population of DMVs retain openings to the cytosol for the duration of infection. Given that viral replication is likely to occur on the cytosolic face of early single-membrane ROs (37, 38), the interior of open DMVs could represent a shielded environment for replication at later stages of infection, with access to the cytosol for virion packing and/or export. A similar enwrapping mechanism could also underlie the formation of the sparse double-membrane structures among abundant single-membrane structures identified in 2D EM investigations of foot-and-mouth disease virus infection (genus *Aphthovirus*) (39) and may represent the means by which DMVs form during infections with *Picornaviridae* more broadly. Evidence for a similar mechanism for DMV biogenesis has been presented for the arterivirus equine arteritis virus and Middle East respiratory syndrome (MERS) coronavirus (order *Nidovirales*), which in this case would occur not through single-membrane intermediate ROs but by direct pairing and curving of the ER membranes (40, 41). While this enwrapping mechanism could represent a general route for DMV RO formation in +RNA viruses, it is intriguing that DMV formation is possible both via enwrapping of Golgi apparatus- or ER-derived single-membrane ROs in the case of picornaviruses and via direct enwrapping of cytosol by ER membranes in the case of nidoviruses. The formation of DMVs from these diverse progenitor membranes could suggest that their development depends primarily on viral machinery or recruited host factors. The cellular functions of these host factors may also provide clues about their role during infection. For instance, LC3 has been found in conjunction with enterovirus ROs (42, 43). In uninfected cells, lipidated LC3 is hypothesized to induce the membrane curvature underlying omegasome formation during autophagy (44), and its recruitment to enterovirus ROs could suggest a similar role in the formation of DMVs, whose development appears to resemble that of autophagosomes.

While direct actors have yet to be established, it is likely that cardiovirus RO formation is dependent upon specific, membrane-associated host factors. PI4P is an essential lipid for the replication of picornaviruses (16, 18, 45) and is generated at the ER or the Golgi apparatus by PI4KA and PI4KB, respectively. Interestingly, PI4Ks are also important for HCV replication, where they appear to play a role in RO formation (19, 46). Expression of a mutated HCV NS5A, whose ability to recruit PI4KA is impaired, has been shown to produce ROs with irregular morphologies (19). A role for PI4Ks during RO biogenesis has also been demonstrated for enteroviruses, as RO formation was delayed under PI4KB inhibition during infections of the PI4KB inhibitor-resistant virus coxsackievirus B3 (CVB3) 3A-H57Y. Under these conditions, ROs were not detected at the peak of viral RNA synthesis, and this process occurred instead on apparently intact Golgi membranes (20). In our study, PI4KA inhibition shifted the subcellular distribution of EMCV dsRNA but did not affect the time postinfection at which ROs were first detected or alter their observed morphology, connectivity with the ER, or competence to support viral RNA synthesis. These different observations in the two genera could arise from differences in the efficiency of the escape mechanism conferred by the specific resistance mutations or alternatively suggest that, unlike for enteroviruses, PI4P does not play a significant role in cardiovirus RO formation. In either case, both mutants were able to (ultimately) produce ROs with normal morphology under PI4K inhibition, showing that any role of high PI4P levels in RO formation can be circumvented by members of both picornavirus genera. PI4P is also required for efficient polyprotein processing in enteroviruses, although this requirement is circumvented in PI4KB inhibitor escape mutants (20, 33, 34). Whether EMCV requires PI4P for polyprotein processing remains to be established.

ROs are believed to confer inherent advantages on the viruses that generate them, but it is unclear whether the different morphologies generated provide specific benefits. Although both single- and double-membrane ROs were found to support cardiovirus RNA synthesis, single-membrane structures predominated during the peak hours of genome replication. While this suggests that DMVs are largely superfluous for RNA synthesis, they may provide other benefits. DMVs could selectively sequester viral products to prevent their detection by cellular innate immune sensors. In the case of enteroviruses, it has been suggested that DMVs may also be utilized for the nonlytic release of viral progeny (47). However, virions were only rarely detected within EMCV DMVs, which likely reflects random incorporation of virus particles and other cytosolic material when DMVs arise by enwrapping. Intriguingly, we found a large proportion of EMCV virions adjacent to ROs, suggesting close spatial coordination between RNA synthesis and capsid assembly. In enteroviruses, viral RNA synthesis and capsid assembly have been shown to be directly coupled, likely through interactions between the RNA replication machinery and the viral structural proteins (48, 49).

Collectively, our data reveal striking similarities between the ROs produced by enteroviruses and cardioviruses and raise the possibility of a conserved mechanism of picornavirus RO biogenesis to form single-membrane structures that can transform into DMVs. In this regard, picornaviruses appear to be unique, as other DMV-forming viruses, like coronaviruses or HCV, generate DMVs directly from the cellular donor membrane. Given that all +RNA viruses to date have been found to produce spherules or DMVs, viruses tend to be classified as producing one or the other RO type. However, while picornaviruses do produce DMVs, RNA synthesis transpires predominantly on the early single-membrane structures. Thus, in addition to the negative-curvature spherules of alphaviruses and flaviviruses and the DMVs formed by coronaviruses and hepaciviruses, positive-curvature single-membrane structures appear to represent a third major form of RO.

## MATERIALS AND METHODS

### Cell lines and reagents.

HeLa R19 cells were maintained in Dulbecco’s modified Eagle’s medium (Gibco) supplemented with 10% fetal calf serum, penicillin, and streptomycin and grown at 37°C in 5% CO_2_. The PI4KA inhibitor A1 (50) was kindly provided by T. Balla (National Institute of Child Health and Human Development, National Institutes of Health, Bethesda, MD) and used at a concentration of 10 nM.

### Virus infections.

Cells were inoculated for 1 h with EMCV wt (strain Mengovirus) or EMCV 3A-A32V (described in reference 26) at an MOI of 50, after which the inoculum was removed and fresh medium (with drug A1 where indicated) was added. At specified time points after infection, cells were prepared for qPCR, viral titer determination, immunofluorescence, or electron microscopy.

### Quantitative PCR.

A NucleoSpin RNA kit (Macherey-Nagel) was used to isolate RNA from lysed cells. Random hexamer primers were used with a TaqMan reverse transcription reagent kit (Roche) to synthesize cDNA. Quantitative PCR was carried out using the LightCycler 480 SYBR green I master kit (Roche) for 45 cycles (5 s at 95°C, 10 s at 60°C, and 20 s at 72°C) on a LightCycler 480 (Roche). Resulting threshold cycle (*C*_*T*_) values were expressed as fold increase with the value at *t* = 2 h set as 1.

### Viral titer determination.

Total virus titers (of intra- and extracellular particles) were determined by freeze-thaw lysis of infected cells and endpoint titration.

### Immunofluorescence microscopy.

Cells were fixed with 3% paraformaldehyde and permeabilized with phosphate-buffered saline (PBS) containing 0.1% Triton X-100. Cells were then labeled with a mouse monoclonal antibody against dsRNA (J2 antibody; English and Scientific Consulting) and secondary Alexa Fluor 488-conjugated goat anti-mouse IgG, alongside a nuclear stain (Hoechst 33342). Imaging was performed using a wide-field DM5500 (Leica) fluorescence microscope.

### Electron microscopy. (i) Metabolic labeling and autoradiography.

Infected cells were preincubated with 10 µg/ml dactinomycin for 1 h to inhibit cellular transcription and then labeled for 45 min with tritiated uridine ([5-^3^H]uridine; 1 mCi/ml) (PerkinElmer) also containing dactinomycin. Cells were then washed several times to remove unincorporated label and fixed for 1 h in 1.5% glutaraldehyde in 0.1 M cacodylate buffer. Mock-infected control samples were similarly prepared. Postfixation consisted of a 1-h incubation with 1% osmium tetroxide in 0.1 M cacodylate buffer followed by a 1-h incubation with 1% uranyl acetate solution. Samples were then washed and dehydrated in ethanol and infiltrated and embedded in epoxy resin LX-112 before polymerization at 60°C. Sections of 50 nm were collected on Formvar-coated EM grids, poststained with lead citrate and uranyl acetate, and then carbon coated and prepared for autoradiography. For this, a thin layer of silver halide photographic emulsion (Ilford L4) was applied in the dark to the cell sections. Samples were incubated in the dark and developed after several weeks for autoradiography as described in reference 20. During incubation, the radiolabeled uridine incorporated during viral RNA synthesis decays, and the beta radiation emitted reduces the silver halide to atomic silver. These events manifest as small, electron-dense grains dispersed around the original radioactive source that can be visualized during EM imaging.

### (ii) High-pressure freezing and freeze substitution.

Cells were high-pressure frozen using an EM PACT2 (Leica). Samples were maintained at −90°C in an AFS2 (Leica) freeze substitution device for 44 h in a solution of 20% H_2_O, 2% osmium tetroxide, and 1% anhydrous glutaraldehyde. A high water content was used as this was found to improve the contrast of RO membranes. The temperature was then raised to 0°C over a period of 22 h through a series of controlled warming phases (identical to those described in reference 21). Samples were washed with acetone, infiltrated with epoxy resin LX-112 (Ladd Research), and polymerized at 60°C. Sections of 70 nm were collected on EM grids and poststained with uranyl acetate and lead citrate. For tomography, thicker sections of 200 nm were collected. Before poststaining, both sides of the EM grid were incubated with 10-nm colloidal gold beads to serve as fiducial markers during tomogram reconstruction.

### (iii) Electron microscopy imaging.

Thin-section (50- or 70-nm) 2D images were collected (binning mode 2) on a Tecnai12 BioTwin or Twin electron microscope at 120 kV using an Eagle 4k slow-scan charge-coupled device (CCD) camera (Thermo Fisher Scientific [formerly FEI]) or OneView 4k high-frame-rate camera (Gatan), respectively. For the semiautomated collection of larger areas, meshes of overlapping images were acquired across the entire region of interest and later reconstituted into a single composite image (as described in reference 51).

### (iv) Electron tomography.

Dual-axis tilt series of 200-nm sections each covering 130 to 140° around the region of interest were collected using an F20 electron microscope (Thermo Fisher Scientific [formerly FEI]) at 200 kV with zero-loss energy filtering using a 2k camera (Gatan) and 1.27-nm pixel size. Automated tilt series acquisition was performed using Xplore3D software (Thermo Fisher Scientific). Tilt series alignment and tomogram reconstruction by weighted back-projection were carried out in IMOD (52) (version 4.7.15). In the process, the gold fiducial markers were digitally erased to be eliminated from the final 3D reconstruction. To determine the sizes of ROs spanning multiple sections, tilt series of the same region across 2 to 5 consecutive sections were collected and combined in IMOD to build tomogram volumes with a *z*-thickness of ~400 to 1,000 nm. The sizes of ROs were estimated by measuring the SMTs, SMVs, and DMVs within reconstructed volumes in IMOD. The maximum widths of SMTs, SMVs, and DMVs were considered the RO diameters. For all calculations, SMTs were approximated to a cylinder, and the inner DMV membrane surface area was considered an approximation of the outer.

To generate models of features of interest, image segmentations were made from tomograms using Amira 6.0.1 (Thermo Fisher Scientific). Before segmentation, tomograms were postprocessed to enhance edges by nonlinear anisotropic diffusion filtering (53) as implemented in IMOD using 5 iterations and *K* = 1,000 and then binned by 2 in *x* and *y*. Membranes were selected in manually masked areas by thresholding and then refined by removing islands and smoothing. Surfaces that were poorly represented (e.g., top and bottom surfaces of vesicles and tubules) due to the missing-wedge effect were manually repaired.
