# Supplementary figures and images for: The Origin, Dynamic Morphology, and PI4P-Independent Formation of Encephalomyocarditis Virus Replication Organelles
Source: mBio. 2018 Apr 17;9(2):e00420-18. doi: 10.1128/mBio.00420-18 (PMC5904412; doi:10.1128/mBio.00420-18)

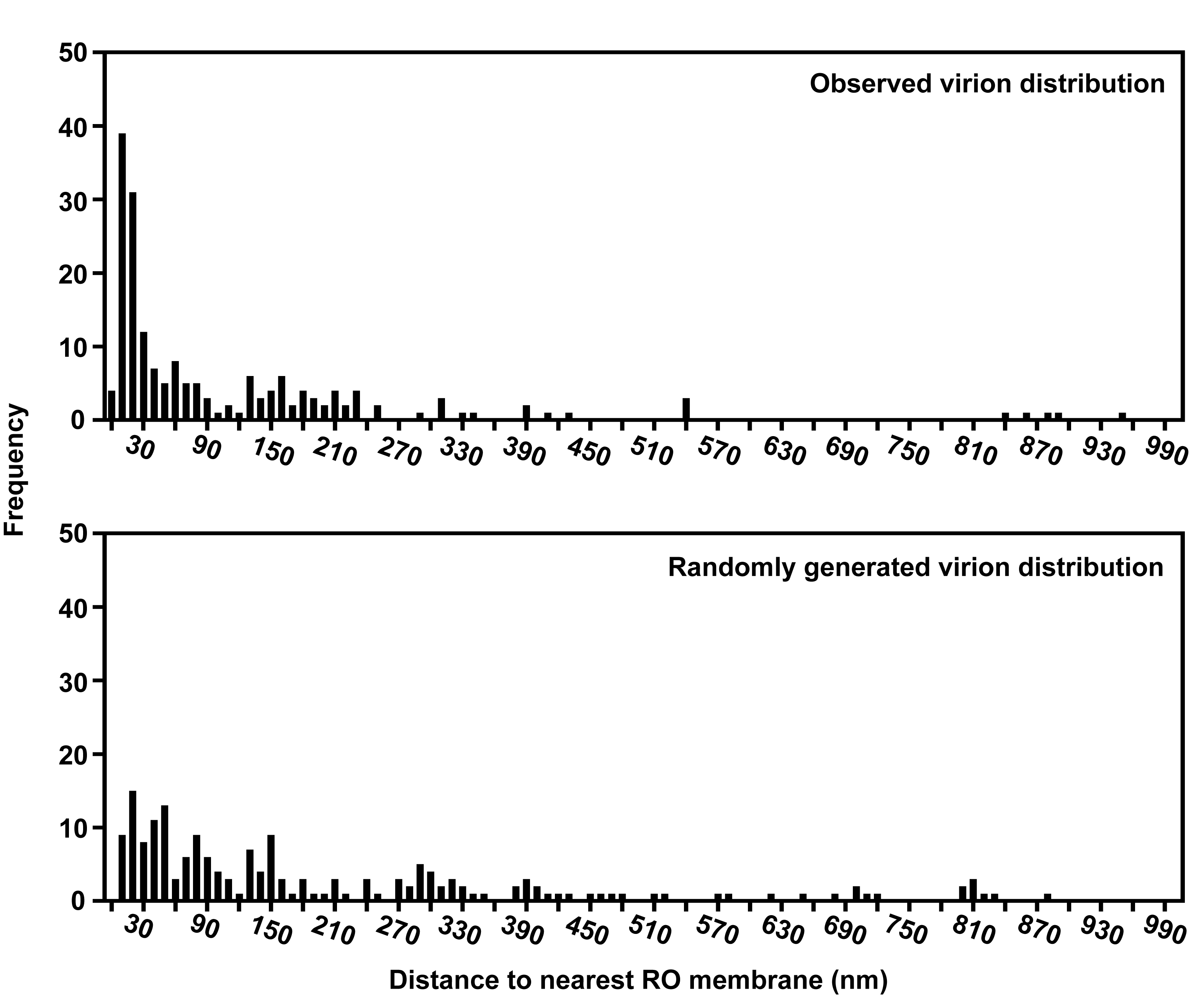

Supplement: FIG S1 [file mbo002183844sf1.tif]

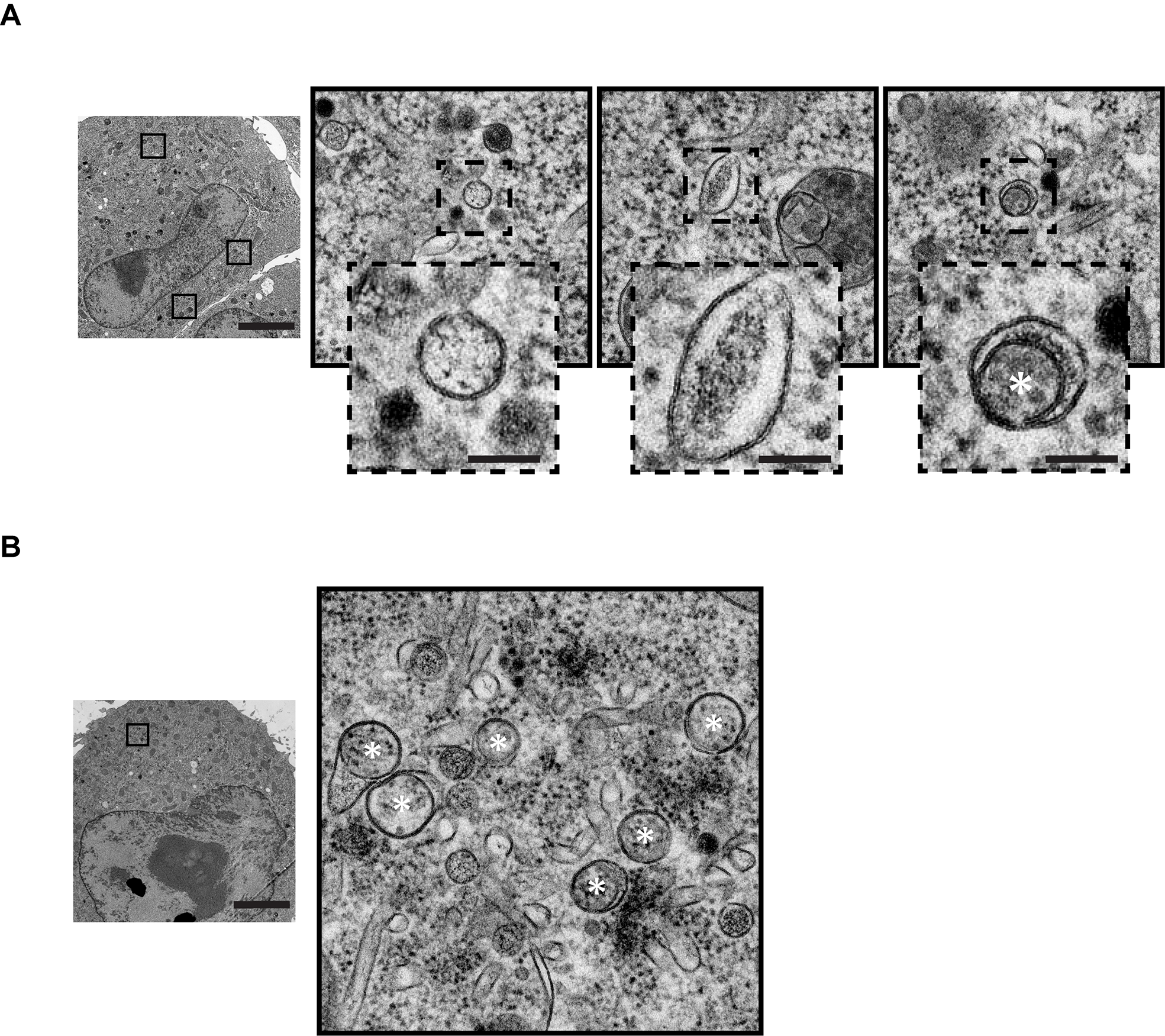

Supplement: FIG S2 [file mbo002183844sf2.tif]

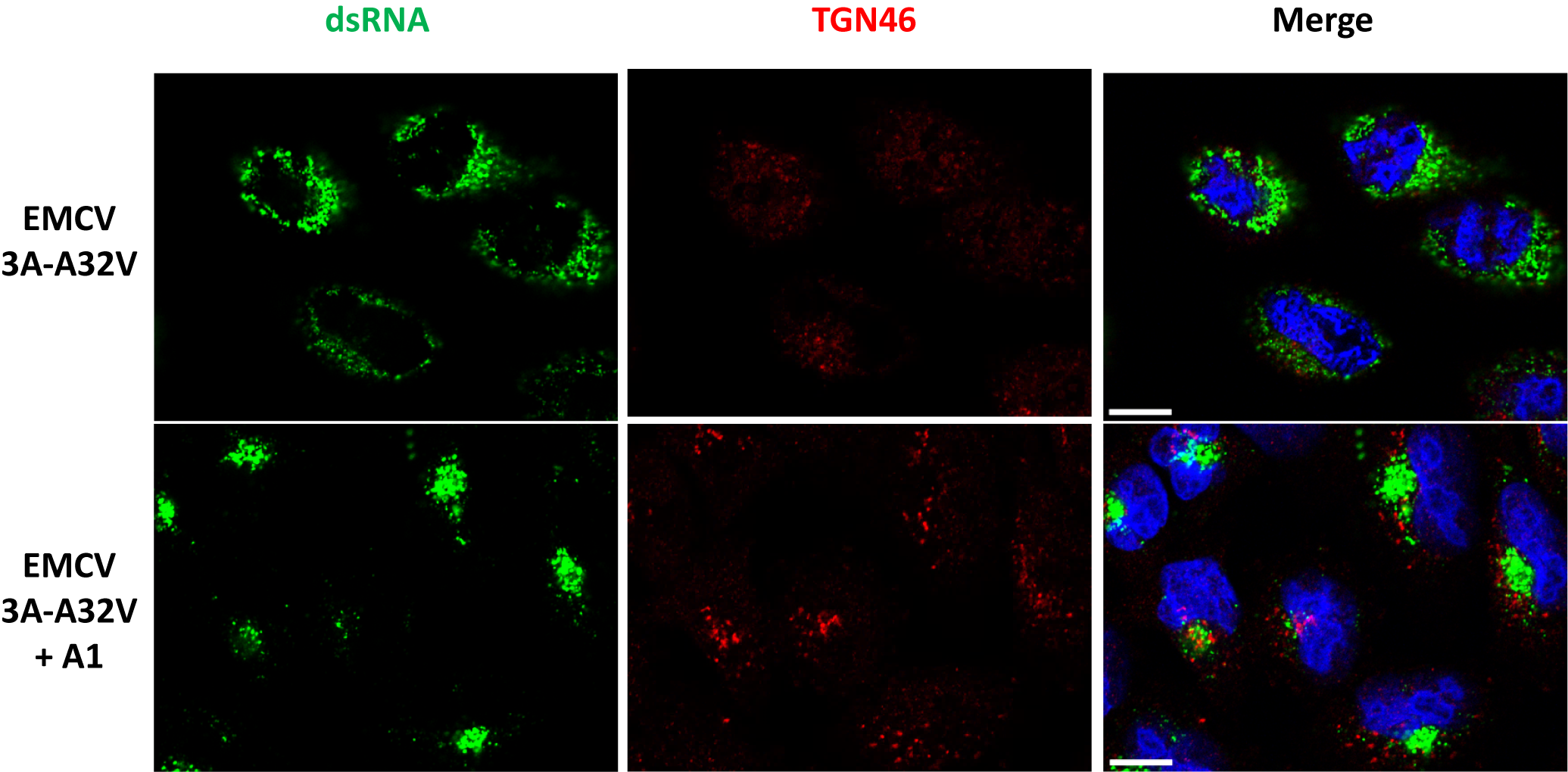

Supplement: FIG S3 [file mbo002183844sf3.tif]
